# PGC-1α activity in nigral dopamine neurons determines vulnerability to α-synuclein

**DOI:** 10.1186/s40478-015-0200-8

**Published:** 2015-04-01

**Authors:** Carine Ciron, Lu Zheng, Wojciech Bobela, Graham W Knott, Teresa C Leone, Daniel P Kelly, Bernard L Schneider

**Affiliations:** Brain Mind Institute, Ecole Polytechnique Fédérale de Lausanne (EPFL), EPFL-SV-BMI-LEN, Station 19, 1015 Lausanne, Switzerland; Centre of Interdisciplinary Electron Microscopy, EPFL, Lausanne, Switzerland; Sanford-Burnham Medical Research Institute, Orlando, FL USA

**Keywords:** PGC-1α, α-synuclein, Parkinson’s disease, Mitochondria, Aging, Neurodegeneration

## Abstract

**Introduction:**

Mitochondrial dysfunction and oxidative stress are critical factors in the pathogenesis of age-dependent neurodegenerative diseases. PGC-1α, a master regulator of mitochondrial biogenesis and cellular antioxidant defense, has emerged as a possible therapeutic target for Parkinson’s disease, with important roles in the function and survival of dopaminergic neurons in the substantia nigra. The objective of this study is to determine if the loss of PGC-1α activity contributes to α-synuclein-induced degeneration.

**Results:**

We explore the vulnerability of PGC-1α null mice to the accumulation of human α-synuclein in nigral neurons, and assess the neuroprotective effect of AAV-mediated PGC-1α expression in this experimental model. Using neuronal cultures derived from these mice, mitochondrial respiration and production of reactive oxygen species are assessed in conditions of human α-synuclein overexpression. We find ultrastructural evidence for abnormal mitochondria and fragmented endoplasmic reticulum in the nigral dopaminergic neurons of PGC-1α null mice. Furthermore, PGC-1α null nigral neurons are more prone to degenerate following overexpression of human α-synuclein, an effect more apparent in male mice. PGC-1α overexpression restores mitochondrial morphology, oxidative stress detoxification and basal respiration, which is consistent with the observed neuroprotection against α-synuclein toxicity in male PGC-1α null mice.

**Conclusions:**

Altogether, our results highlight an important role for PGC-1α in controlling the mitochondrial function of nigral neurons accumulating α-synuclein, which may be critical for gender-dependent vulnerability to Parkinson’s disease.

**Electronic supplementary material:**

The online version of this article (doi:10.1186/s40478-015-0200-8) contains supplementary material, which is available to authorized users.

## Introduction

In Parkinson’s disease (PD), the age-dependent loss of dopaminergic neurons in the *substantia nigra pars compacta* (SNpc) has been linked to mitochondrial dysfunction. Alterations of the electron transport chain (ETC) activity can be caused by environmental factors, such as accidental exposure to 1-methyl-4-phenyl-1,2,3,4-tetrahydropyridine (MPTP) [1,2]. Similarly, a parkinsonian syndrome characterized by selective nigral degeneration can be induced in rodents following chronic mitochondrial intoxication with rotenone, which impairs mitochondrial complex I activity [3]. The aging process may also cause the accumulation of genetic defects in mitochondrial DNA, thereby contributing to neurodegeneration in PD [4,5].

The link between PD and mitochondrial defects has gained further support when PD-associated genetic factors, such as α-synuclein (aSyn), LRRK2, parkin, PINK1 and DJ-1, were shown to be involved in the function and turnover of mitochondria. Notably, the ubiquitin ligase parkin and the mitochondrial kinase PINK1, which are linked to autosomal recessive juvenile PD [6,7], control the autophagic clearance of defective mitochondria [8,9]. DJ-1 maintains proper mitochondrial function in response to oxidative stress [10].

Multiplications or point mutations in the gene encoding aSyn, an abundant presynaptic protein, are associated with autosomal dominant familial PD. Accumulation or mutations of the aSyn protein increase its propensity to adopt a β-sheet conformation, thereby producing oligomers and fibrils that accumulate in Lewy bodies. Alpha-synuclein interacts with the mitochondrial outer membrane, inducing mitochondrial fragmentation [11-13]. Transgenic mice expressing human aSyn show pathogenic impairments of the mitochondrial function [14,15] and conversely, aSyn-null mice often display increased resistance to MPTP [16,17].

Factors modulating mitochondrial activity have emerged as novel therapeutic targets in PD. Peroxisome proliferator-activated receptor gamma coactivator-1α (PGC-1α) is a master transcriptional regulator of cell metabolism, controlling the expression of nuclear genes implicated in mitochondrial biogenesis and resistance to oxidative stress [18]. In neuronal cultures from the ventral midbrain, PGC-1α increases mitochondrial mass and basal respiration [19], and transgenic mice overexpressing PGC-1α in dopaminergic neurons are more resistant to MPTP [20]. A meta-analysis of gene expression changes in the SN of human PD patients has revealed underexpression of PGC-1α target genes implicated in mitochondrial function, consistent with a loss of PGC-1α activity that may play a key role in disease pathogenesis [21]. Furthermore, reduced PGC-1α activity has recently been shown to enhance aSyn oligomerization, which in turn downregulates PGC-1α expression [22].

Unexpectedly, chronic supraphysiologic expression of PGC-1α selectively impairs dopaminergic function in adult rats and mice [19,23]. Nevertheless, it is also important to explore if decreased PGC-1α activity, which presumably occurs in the aged, parkinsonian brain [24], is linked to neuronal loss in sporadic PD. Although PGC-1α null mice do not spontaneously develop a parkinsonian syndrome, they display higher sensitivity to oxidative stress and excitotoxic injuries [18]. Here, we hypothesized that the loss of PGC-1α activity may increase the vulnerability to aSyn via perturbations of the mitochondrial activity and reduced detoxification of reactive oxygen species (ROS). To address this possibility, we used null mice with disrupted expression of full-length PGC-1α (PGC1α-KO) [25]. We report that nigral dopaminergic neurons in PGC1α-KO mice show abnormal mitochondria and fragmented endoplasmic reticulum (ER). Furthermore, these neurons are more prone to degenerate following overexpression of human aSyn. This effect is more apparent in male PGC1α-KO mice and can be rescued by AAV-mediated expression of PGC-1α. Altogether, our results highlight a gender-dependent role of PGC-1α in neuronal vulnerability to aSyn.

## Materials and methods

### Plasmid construction

Human wild-type (WT) aSyn (nucleotides 46–520, GeneBank accession no. NM_000345) and full-length mouse PGC-1α (nucleotides 35–2428, GeneBank accession no. BC066868) were inserted into the pAAV-pgk-MCS backbone, modified from the serotype 2 pAAV-cmv-MCS (Agilent, La Jolla, CA, USA) using standard cloning procedures. To enhance transgene expression, regulatory elements including an optimized kozak consensus sequence and the Woodchuck hepatitis virus Post-transcriptional Regulatory Element (WPRE), were inserted into pAAV-pgk-MCS, leading to our 2nd generation vector which was used for enhanced expression of human WT aSyn [26]. The pAAV-cmv-MitoDsRed plasmid was generated by subcloning the coding sequence for DsRed2 fused to a mitochondrial localization signal into the pAAV-cmv-MCS backbone.

### Recombinant AAV production and titration

Recombinant pseudotyped rAAV2/6 were produced, purified and titrated as described previously [19]. A stuffer sequence was included in the plasmid pAAV-pgk-MCS to generate a non-coding vector with a comparable genome size. The measured titers were as follows: AAV-pgk-aSynWT 1.1 × 10^10^ TU/ml; AAV-pgk-Kz-aSynWT-WPRE 2.1 × 10^10^ TU/ml; AAV-pgk-MCS (non-coding vector) 7.8 × 10^10^ TU/ml; AAV-pgk-PGC-1α 7.4 × 10^10^ TU/ml. Except for expression of mitoDsRed, serotype 6 pseudotyped particles were used in the present study. The AAV-cmv-MitoDsRed vector was packaged into serotype 8 AAV particles as described previously [27], with a titer of 9.4 × 10^12^ VG/ml. The serotype 8 was preferred in this case to avoid retrograde transduction of neurons in the striatum [27].

### Stereotaxic unilateral injection into the SNpc of mice

Young adult (6 and 8 weeks old) C57BL/6J (Charles River Laboratories, France) and PGC1α-KO mice were housed in a 12 hrs light/dark cycle, with *ad libitum* access to food and water, in accordance with Swiss legislation and the European Community Council directive (86/609/EEC) for the care and use of laboratory animals. The line of PGC1α-KO mice was generated and first described [25] by the Laboratory of Dr. Daniel Kelly at Sanford-Burnham Medical Research Institute (Orlando, FL) and backcrossed into C57BL/6J for 10 generations. For the present study, the PGC1α-KO mice were bred at EPFL. For stereotaxic injections, the animals were deeply anesthetized with a mixture of xylazine/ketamine and placed in the stereotaxic frame (David Kopf Instruments, Tujunga, CA, USA). 1 μl of viral preparation, was injected in the right brain hemisphere using a 10 μl Hamilton syringe with a 34-gauge blunt tip needle at a speed of 0.2 μl/min, using an automatic pump (CMA Microdialysis, Sweden). The total injected vector load was 1 × 10^7^ TU for a single vector injection, and 1.5 × 10^7^ TU when two vectors were co-injected. The needle was left in place for an additional 5 min before being slowly withdrawn. The SNpc was targeted at the following coordinates: anterio-posterior (AP) -2.9 mm and mediolateral (ML) 1.3 mm relative to bregma, dorsolateral (DV) -4.2 mm relative to skull surface.

For injection of the AAV8-cmv-MitoDsRed in the SNpc of 10-month old mice, we used the following coordinates: antero-posterior (AP) -2.9 mm and mediolateral (ML) 1.6 mm relative to bregma, dorsolateral (DV) -4.9 mm relative to skull surface.

### Primary neuronal culture from mouse cerebral cortex

The protocol used for the preparation of the primary neuronal cultures from mouse cerebral cortex was adapted from reference [19]. Cortices of embryonic brains (E16.5) were dissected under a microscope and kept in cold Hank’s buffered salt solution (HBSS) w/o Ca^2+^ and Mg^2+^. Tissues were dissociated by gentle trituration through a Pasteur pipette. Cells were resuspended in MEM medium supplemented with 10% heat inactivated horse serum, GlutaMAX 1X and 1% Pen-Strep and filtered (0.22 μm). The cell concentration and the viability were determined using a classic dye exclusion method (Trypan Blue). Neurons were seeded onto 24-well culture plates precoated with poly-D-lysine (100 mg/ml) and laminin (mg/ml) at a density of 1 × 10^5^ cells per well. Plates were maintained at 37°C in a humidified atmosphere of 5% CO_2_. Three hours after plating, medium was removed and replaced by Neurobasal medium supplemented with B27 (1X), GlutaMAX (1X) and 1% Pen-Strep. Seven-day-old cultures were used for AAV infections. For AAV-mediated transduction, each 24-well was infected with a viral dose of 6 × 10^6^ TU.

### Measurements of oxygen consumption

Oxygen measurements were made using the XF96 Extracellular Flux Analyzer (Seahorse Bioscience, Billerica, MA). Primary cortical neurons were seeded in pre-coated XF96-well microplates at 4.0 × 10^4^ cells per well and cultured for 5 days in regular conditions. Neurons were then transduced with AAV2/6 vectors at a total dose of 1 × 10^6^ TU. Seven days after infection, cells were used for analysis of oxygen consumption. Two days before measurement, 75% of the culture medium was replaced by Neurobasal A medium (GIBCO, 005-0128DJ) supplemented with 2 mM glucose, 25 mM pyruvate, 100 U/ml Penicillin-Streptomycin, 2 mM glutamax and B27. One day before measurement the cartridge with electrodes was subjected to automatic calibration. Basal oxygen consumption rate (OCR) was measured four times over 30 min. Cells in separate wells were next exposed to either oligomycin (5 μM) or CCCP (10 μM) for 35 min and respiration rate was measured five times. The average of the first two measures of CCCP-induced OCR were used for calculation of the spare respiratory capacity. Calculations of oligomycin-resistant respiration and percentage of OCR used for ATP production were based on the average of five OCR measurements in oligomycin-treated cells.

### Immunohistological analyses and quantification of nigrostriatal lesions

Mice were sacrificed and tissue processed as described previously [19]. Primary antibodies used in this study were anti-TH (Rabbit IgG, 1:1000; AB152, Chemicon and mouse IgG for triple staining, 1:800; Sigma-Aldrich), anti-cleaved Caspase 3 (Rabbit IgG, 1:500; Cell Signaling), anti-aSyn (Sheep IgG, 1:400; Chemicon), anti-PGC-1α (rabbit IgG, 1:1000; generous gift from Daniel Kelly, Sanford-Burnham Medical Research Institute, Orlando, FL, USA), anti-4-Hydroxynonenal (HNE) (Rabbit IgG, 1:1000; Alexis). For fluorescence labeling, we used secondary antibodies conjugated to Alexa Fluor-488 (1:500; Invitrogen), Cy3 (1:1000; Jackson ImmunoResearch), AMCA (1:100; Vector Laboratories). For bright-field microscopy, we used biotinylated goat anti-rabbit secondary antibody (1:200; Vector Laboratories).

To analyze mitoDsRed-labeled mitochondria in the dorsolateral region of the striatum of 10-month old mice, 4 weeks after injection of the AAV-mitoDsRed vector, coronal brain sections were subjected to confocal microscopy analysis using a Zeiss LSM700 UP2 microscope with a 40x objective. Three images were arbitrarily taken from three different brain sections of each animal, and the density of mitochondria was measured by normalizing the number of discrete mitoDsRed signals to the dopaminergic axon area, as determined by the surface of TH immunoreactivity. Images were processed using ImageJ, Huang and Triangle were selected as proper algorithms to set threshold for mitochondria and axons, respectively.

Stereological estimates of Nissl-positive and TH-positive nigral neurons were obtained as described previously [19]. The extent of striatal dopaminergic innervation was measured by determining the optical density for TH on DAB-immunostained brain slices regularly distributed over the entire striatum. All immunohistochemical evaluations were performed in a blinded manner.

### Transmission electron microscopy

Adult 10 months-old mice were perfused via the heart with 2.5% glutaraldehyde and 2% paraformaldehyde in 0.1 M phosphate buffer at pH 7.4. After 2 hrs the brains were removed and vibratome sectioned coronally, at 80 μm thickness, at the level of the SN. The sections were washed in 0.1 M cacodylate buffer, post-fixed in 1.5% potassium ferrocyanide, followed by 1% osmium tetroxide, and then 1% uranyl acetate, for 40 min in each solution. They were then dehydrated in a graded alcohol series, embedded in Durcupan resin (Fluka, Switzerland) and then mounted between two glass microscope slides before being hardened at 60°C for 48 hrs. The region of interest was selected using a stereomicroscope and transmitted light to image the brain sections. Using a razor blade, the selected part was then removed and glued to a blank resin block for further trimming and sectioning. Ultrathin sections were cut at 50 nm and collected onto formvar coated nickel slot grids, and imaged at 80 kV in a transmission electron microscope (Tecnai Spirit FEI), and images collected with a CCD camera at a resolution of approximately 2 nm per pixel (FEI Eagle).

From each mouse, single thin sections of the SN and VTA were systematically scanned, and every neuronal cell body in which a nucleus could be seen was imaged. In total, 79, 89 and 113 cell bodies were imaged in the SN of WT, PGC1α-KO and PGC1α Inj conditions, respectively. Then electron microscopy images were analyzed using ImageJ software. Three mice were analyzed per group, corresponding to a total number of 2729, 3527 and 2544 mitochondria in WT, PGC1α-KO and PGC1α Inj, respectively. In the VTA, 51 and 95 cell bodies were imaged in WT and PGC1α-KO mice, respectively. Three mice were similarly analyzed per group, corresponding to a total number of 4110 and 7265 mitochondria in WT and PGC1α-KO mice, respectively.

### RNA extraction and RT-qPCR

To determine the level of PGC-1α and PGC-1β mRNA in the SN, age-matched WT, PGC1α-KO and AAV-PGC-1α-injected mice were sacrificed by decapitation. The AAV vector encoding mouse PGC-1α was injected 3 weeks before animal sacrifice (n = 3 per group). In each mouse, the SN was rapidly dissected and transcript abundance measured by reverse transcription-quantitative PCR (RT-qPCR). Total RNA was isolated with an RNAeasy Mini Kit (Qiagen). cDNA was prepared using a Omniscript Reverse Transcription Kit. Briefly, total RNA (50 ng) was reverse transcribed in a final volume of 20 μl with OligodT primers at 37°C for 1 hr according to the manufacturer’s instructions. The expression levels of PGC-1α and PGC-1β were measured by RT-qPCR using SybrGreen assays. The genes ACTB and B2M were used as endogenous controls. We used Quantitect Primer Assays (Qiagen) to quantify expression of these 4 genes. Each assay was run in duplicate, with the Rotor-Gene Sybr Green PCR Kit (Qiagen) on a Rotor-Gene Cycler using the following cycling conditions: 5 min at 95°C, then 5 sec at 95°C and 10 sec at 60°C for 40 cycles. Each replicate’s cycle threshold (Ct) was normalized to the average Ct of the two endogenous controls on a per sample basis. The comparative Ct method was used to calculate relative levels of PGC-1α and PGC-1β expression, as described in [28].

### Measurements of H_2_O_2_ concentration

Primary cortical neurons were seeded in precoated 24-well plates at 1x10^5^ cells per well and cultured for 7 days in 37°C/5% CO_2_ incubators. Neurons were co-infected with AAV2/6 vectors at a dose of 6 × 10^6^ TU for each vector. Measurements of H_2_O_2_ concentration in the culture medium were made using Amplex Red Hydrogen Peroxide/Peroxidase Assay Kit (Invitrogen). Fifty μl of the standard curve samples and experimental samples were added to individual wells of a microplate, as well as 50 μl of the Amplex Red reagent/HRP working solution. After 30 min of incubation at room temperature, plates were analyzed on a fluorometer (Tecan Safire2 microplate reader) using wavelengths at 530 nm for excitation and 585 nm for emission.

### Statistical analysis

Data are expressed as average ± standard error of the mean (SEM). Statistical analysis was performed using the Statistica software (StatSoft Inc., OK, USA). The alpha level of significance was set at 0.05. Applied statistical tests are indicated in the Figure legends.

## Results

### Transcriptional activity of genes related to mitochondrial function is changed in PGC1α-KO mice

To assess how PGC-1α activity impacts on nigral dopaminergic neurons, we used PGC1α-KO mice obtained by homologous recombination, with an incomplete targeting of the mouse PGC-1α coding sequence [25]. Although the expression of full length PGC-1α is almost completely extinguished in homozygous mice, they retain the potential to produce a truncated form of the protein (amino acids 1–254), named NT-PGC-1α254, and which is functionally similar to the natural splice variant NT-PGC-1α [29,30].

PGC1α-KO mice were compared to control wild-type (WT) C57BL/6J mice, from the same genetic background. In order to perform rescue experiments and restore PGC-1α activity, we designed an AAV2/6 vector for constitutive expression of PGC-1α following injection in the adult SNpc.

**Figure 1:**
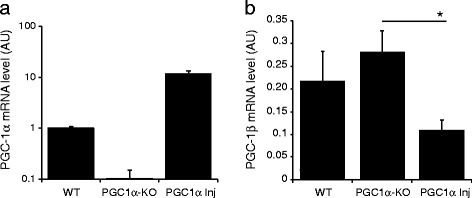
**Expression of PGC-1α and PGC-1β in PGC1α-KO mice. (a)** Level of PGC-1α mRNA in the SN of WT mice, PGC-1α KO mice (PGC1α-KO) and from PGC1α-KO mice injected with a vector encoding PGC-1α (PGC1α Inj) at 1 month post-injection. Values are expressed in arbitrary units (AU). WT n = 2; PGC1α-KO n = 5; PGC1α Inj n = 3. **(b)** Level of PGC-1β mRNA in the SN. Values are expressed in arbitrary units (AU). WT n = 3; PGC1α-KO n = 3; PGC1α Inj n = 3. Statistical analysis: one-way ANOVA with Newman-Keuls post-hoc test; *p < 0.05.

First, we assessed PGC-1α expression in the SNpc (Figure 1a). RT-qPCR revealed in adult PGC1α-KO mice a 9.7-fold reduction in the level of the PGC-1α transcript (0.10 ± 0.04 Arbitrary Units (AU)), as compared to WT mice (1.0 ± 0.1 AU). Of note, PCR primers were designed to amplify a region between exon 5 and exon 6 of the PGC-1α gene transcript, which is predicted to be non-transcribed in PGC1α-KO mice [25]. In PGC1α-KO mice injected with 1x10^7^ TU of AAV2/6 vector expressing PGC-1α (PGC1α Inj), we measured at three weeks after injection a 11.8-fold increase in the abundance of the PGC-1α transcript in the SNpc (11.8 ± 1.7 AU), as compared to endogenous level in WT mice.

Next, we sought to explore if the loss of PGC-1α activity could modify expression of PGC-1β, a closely related member of the peroxisome proliferator-activated receptor gamma co-activator family. PGC-1β also controls mitochondrial capacity, and could therefore compensate for the lack of PGC-1α activity [31]. In the SNpc of PGC1α-KO mice, we measured a modest, non-significant increase in the level of PGC-1β expression (0.28 ± 0.05 AU), when compared to WT mice (0.21 ± 0.06 AU; Figure 1b). Conversely, the abundance of the PGC-1β transcript was significantly reduced to 0.11 ± 0.02 AU when PGC-1α was overexpressed three weeks post-injection of the AAV-PGC-1α vector.

### PGC1α-KO nigral dopaminergic neurons accumulate large-sized mitochondria and display a disorganized endoplasmic reticulum

Next, we explored by electron microscopy how the loss of PGC-1α affects mitochondria in nigral dopaminergic neurons. We analyzed the ultrastructure of organelles in the soma of neurons located in the SNpc and ventral tegmental area (VTA) of 10 month-old mice. PGC1α-KO mice were compared to WT mice, and PGC1α-KO mice at 4 months after intranigral injection of the AAV-PGC-1α vector to restore PGC-1α activity in the dopaminergic neurons (PGC1α Inj).

**Figure 2:**
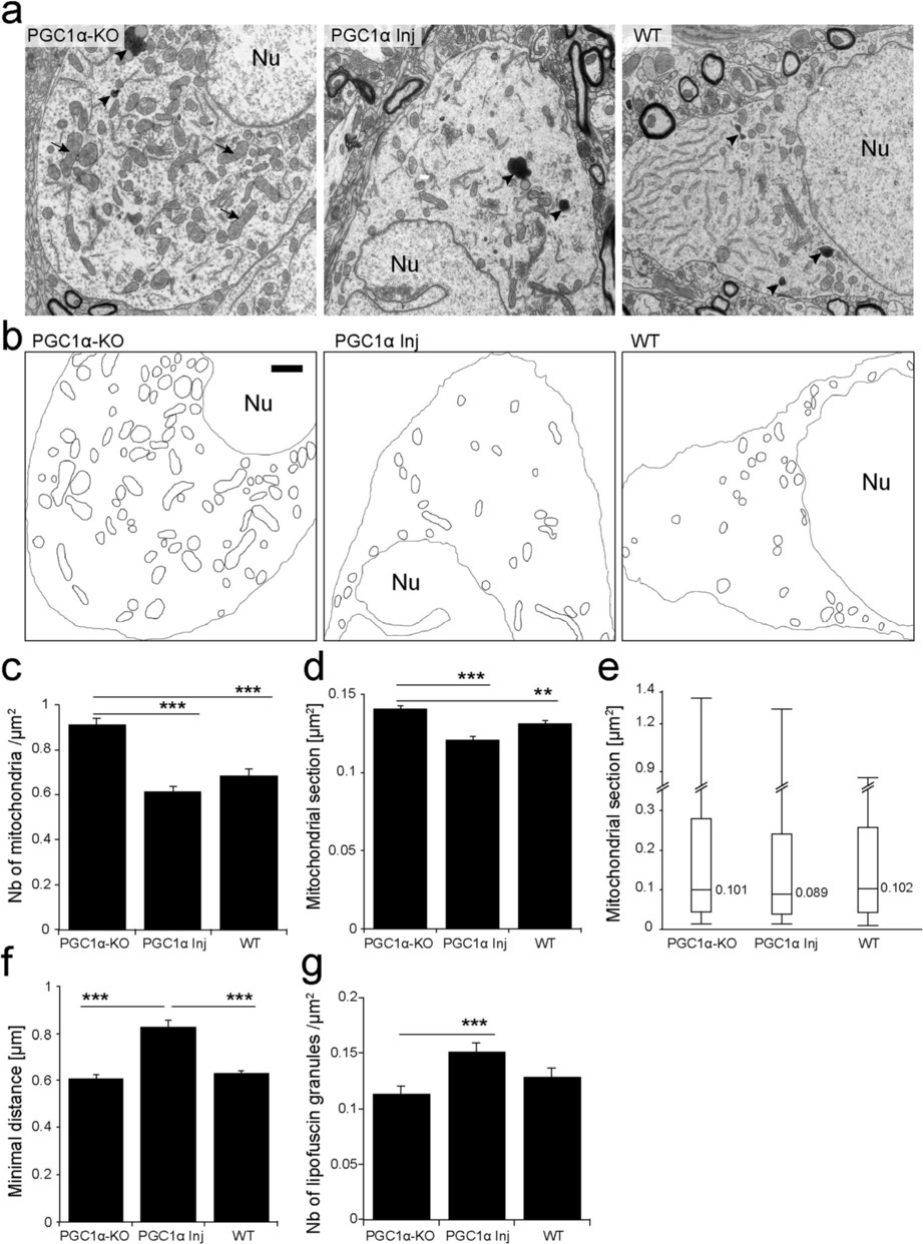
**PGC-1α reduces the number of mitochondria and rescues the abnormal mitochondrial phenotype observed in the SNpc of PGC1α-KO mice. (a)** Electron micrographs of neuronal soma in the SNpc of 10 month-old PGC1α-KO mice, PGC1α-KO mice injected with a vector encoding PGC-1α (PGC1α Inj) and WT mice. Note the presence of lipofuscin granules (black arrowheads) and giant mitochondria with disorganized *cristae* (black arrows). Nu indicates the neuronal nucleus (Nu). **(b)** Mitochondria are outlined with black lines. Neuronal nuclei and membranes are outlined with a grey line to indicate the limits of the neuronal cytosol. Note the increase in the density of mitochondrial clusters in PGC1α-KO mice. Scale bar: 1 μm. **(c)** Quantification of mitochondrial density reveals a significant increase in PGC1α-KO mice compared to the other groups. **(d)** Average area of mitochondria. Note the increased size in PGC1α-KO mice, compared to PGC1α Inj and WT mice. **(e)** Box and whisker plots showing the distribution of mitochondrial size in the SNpc of PGC1α-KO, PGC1α Inj and WT mice. The thick line represents the median and the box indicates the 10th and the 90th percentiles. Whiskers show the extreme values for each group. Note the presence of abnormal, enlarged mitochondria in PGC1α-KO mice. **(f)** Nearest neighbor analysis of mitochondrial distribution in the neuronal cytosol, demonstrating reduced clustering in PGC1α Inj mice. **(g)** Density of lipofuscin granules, which is significantly increased in the PGC1α Inj group. Statistical analysis: one-way ANOVA with Newman-Keuls post-hoc test; (C,F,G): WT: n = 79 neurons; PGC1α-KO: n = 89 neurons; PGC1α Inj: n = 113 neurons. (D,E): WT: n = 2729 mitochondria; PGC1α-KO: n = 3527; PGC1α Inj: n = 2544; **p < 0.01, ***p < 0.001. Micrographs were obtained from 3 animals in each group.

In the SNpc, the density of mitochondria in the neuronal soma was increased in PGC1α-KO mice (0.91 ± 0.03 mitochondria per μm^2^), as compared to WT animals (0.69 ± 0.03 mitochondria per μm^2^; Figure 2a to c). We also found that the average mitochondrial size was significantly larger in dopaminergic neurons of PGC1α-KO mice (mitochondrial area: 0.140 ± 0.002 μm^2^), than in WT mice (0.131 ± 0.002 μm^2^; Figure 2d). Strikingly, giant mitochondria (size >1 μm^2^) were observed in the neuronal soma of PGC1α-KO mice, while they were absent in aged WT mice (Figure 2e). The abnormal presence of giant mitochondria may indicate impaired mitochondrial dynamics and high levels of oxidative stress in PGC1α-KO mice. Remarkably, most of these abnormal mitochondrial features were corrected in the SNpc of mice injected with the AAV-PGC-1α vector. As compared to PGC1α-KO mice, the density of mitochondria in the neuronal cytosol was significantly decreased to 0.62 ± 0.02 mitochondria per μm^2^ in the PGC1α Inj group, a value similar to WT mice (Figure 2c). Similarly, the average size of mitochondrial section was reduced to 0.121 ± 0.002 μm^2^ (Figure 2d). Although some giant mitochondria were still observed in PGC1α Inj mice, the median size was reduced to 0.089 μm^2^ in the PGC1α Inj group, versus 0.101 μm^2^ and 0.102 μm^2^ in PGC1α-KO and WT, respectively (Figure 2e). Overall, the expression of PGC-1α in adult PGC1α-KO mice is associated with the presence of small-sized mitochondria in the neuronal soma. These organelles are more sparsely and homogenously distributed in the cytosol, as indicated by larger minimal distance between mitochondria, measured using the nearest neighbor method (Figure 2f). As compared to PGC1α-KO (0.61 ± 0.02 μm) and WT (0.63 ± 0.01 μm) mice, the average minimal distance between mitochondria was significantly increased to 0.83 ± 0.03 μm following injection of the AAV-PGC-1α vector. Restored expression of PGC-1α in adult PGC1α-KO mice prevented mitochondrial clustering, which is often associated with mitochondrial dysfunction and cell death [32].

To determine if the loss of PGC-1α activity leads to overall changes in the number of mitochondria in nigral dopaminergic neurons, we assessed mitochondrial density in the axonal projections located inside the striatum. An AAV8 vector encoding the mitochondria-targeted marker mitoDsRed was injected in the SNpc of 10 months-old mice, and the density of mitoDsRed-positive organelles was quantified in the dorsolateral part of the striatum (Additional file 1: Figure S1). Mitochondrial accumulation of mitoDsRed was found in the majority of TH-positive dopaminergic neurons in the SNpc (Additional file 1: Figure S1a), and labeled organelles were distributed throughout the dorsolateral striatum (Additional file 1: Figure S1b). Quantification of the density of discrete labeled punctae in the striatum did not reveal any significant difference between PGC1α-KO mice and WT controls (Additional file 1: Figure S1c). As the vast majority of mitochondria are expected to reside within the axonal compartment, there is likely no overall change in mitochondria density in the nigral dopaminergic neurons of PGC1α-KO mice. Therefore, the increased number of enlarged mitochondria present in the soma of these neurons reflects impaired mitochondrial dynamics caused by the loss of PGC-1α expression.

Overexpression of PGC-1α also induced the accumulation of lipofuscin granules in nigral neurons (Figure 2g). The measured density of lipofuscin granules in the neuronal cytosol of PGC Inj animals was 0.15 ± 0.01 granules/μm^2^, significantly increased with respect to PGC1α-KO mice (0.11 ± 0.01 granules/μm^2^). Lipofuscin is composed of non-degradable, electron-dense intralysosomal material, mainly oxidized proteins and lipids [33], the accumulation of which is caused in large part by the deposition of autophagocytosed mitochondria. Lipofuscin is therefore considered as an indicator of mitophagy [34,35], and its accumulation might indicate enhanced mitochondrial turnover in the nigral neurons of mice injected with the AAV-PGC-1α vector.

In the same animals, we explored if similar changes in mitochondrial morphology were present in the VTA, a brain region that contains dopaminergic neurons resistant to PD pathology. Ultrastructural analysis of neuronal cells inside the VTA did not reveal any overt difference between WT and PGC1α-KO mice, neither in mitochondrial density, nor in the intracellular distribution of mitochondria (Additional file 2: Figure S2). Notably, giant mitochondria were not observed in the VTA of PGC1α-KO mice. Instead, the average surface of mitochondrial sections in PGC1α-KO mice was even significantly smaller than in WT (0.133 ± 0.001 vs 0.151 ± 0.001). The contrasted effects observed in the SN and VTA underline the selective vulnerability of nigral dopaminergic neurons to changes in PGC-1α activity.

### PGC1α-KO nigral dopaminergic neurons display a fragmented endoplasmic reticulum, with a reduction in the number of ER-mitochondria contacts

**Figure 3:**
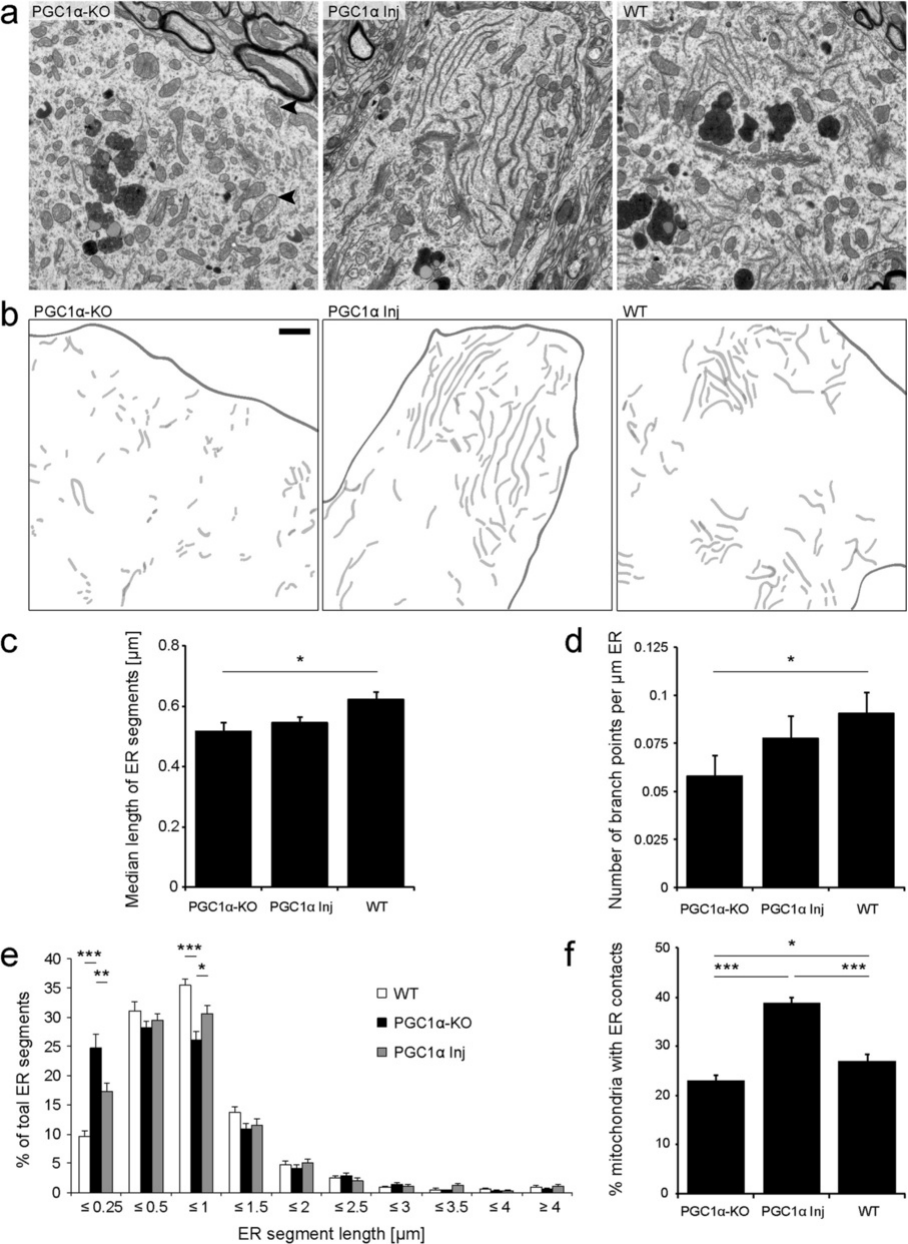
**Expression of PGC-1α rescues ER morphology in PGC1α-KO mice, and increases the number of mitochondrial contacts with ER. (a)** Electron micrographs of neuronal soma in the SNpc of PGC1α-KO, PGC1α Inj and WT mice. Black arrowheads indicate the presence of giant mitochondria with disorganized cristae. **(b)** ER cisternae are colored in light gray. The cell membrane at the border of the neuronal cytosol is outlined. Note that PGC1α-KO mice display a disorganized and fragmented ER. In WT and PGC1α Inj mice, normal ER stacks are observed. Scale bar: 1 μm. **(c,d)** Quantification of the median length of ER profiles and number of branch points per μm of ER. **(e)** Relative length distribution of the ER segments in individual neurons from WT, PGC1α-KO and PGC1α Inj mice. Note the overall fragmentation of the ER in neurons from PGC1α-KO mice. Statistical analysis for c-d: one-way ANOVA with Newman-Keuls post-hoc test; WT: n = 51 neurons; PGC1α-KO: n = 51 neurons; PGC1α Inj: n = 60 neurons **(f)** Percentage of mitochondria having membrane contacts with ER. Note that PGC-1α significant increases the proportion of mitochondria with ER contacts. Statistical analysis: one-way ANOVA with Newman-Keuls post-hoc test; WT: n = 79 neurons; PGC1α-KO: n = 89 neurons; PGC1α Inj: n = 113 neurons; *p < 0.05, **p < 0.001 and ***p < 0.001. Micrographs were obtained from 3 animals in each group.

As functional interactions exist between mitochondria and ER, we examined ER morphology in the soma of dopaminergic neurons in the SNpc (Figure 3). WT mice displayed a normal, parallel-organized ER stack. In stark contrast, the ER of PGC1α-KO mice appeared disorganized and fragmented (Figure 3a and b). In PGC1α-KO mice, we measured in neuronal cells a significant reduction in the median length of ER segments and a significant decrease in the number of ER branches compared to WT animals (Figure 3c and d). By analyzing more closely the size distribution of ER segments, we found a significant increase in the percentage of small ER segments with a length ≤0.25 μm, and a corresponding decrease in ER segments between 0.5 and 1 μm in PGC1α-KO mice compared to WT mice (Figure 3e). Both of these defects were significantly rescued by AAV-mediated expression of PGC-1α (Figure 3e). Next, we examined membrane contacts between mitochondria and ER (Figure 3f). Theses interactions are crucial in a number of physiological processes, including mitochondrial function, mitochondrial biogenesis, lipid metabolism, Ca^2+^ signaling and cell death [36]. As compared to WT and PGC1α-KO mice, we observed a significant increase in the proportion of mitochondria making membrane contacts with the ER in animals injected with the AAV-PGC-1α vector, further highlighting the role of PGC-1α in controlling ER/mitochondria interactions.

Overall, nigral neurons of PGC1α-KO mice show pronounced and specific perturbations of mitochondrial and ER morphology, which reflect profound organelle dysfunctions. Considering the possible implication of reduced PGC-1α activity in aging, we next sought to investigate whether PGC1α-KO mice display increased vulnerability to the accumulation of human aSyn, a key actor in PD pathology.

### Reduced PGC-1α activity increases neuronal vulnerability to α-synuclein toxicity

**Figure 4:**
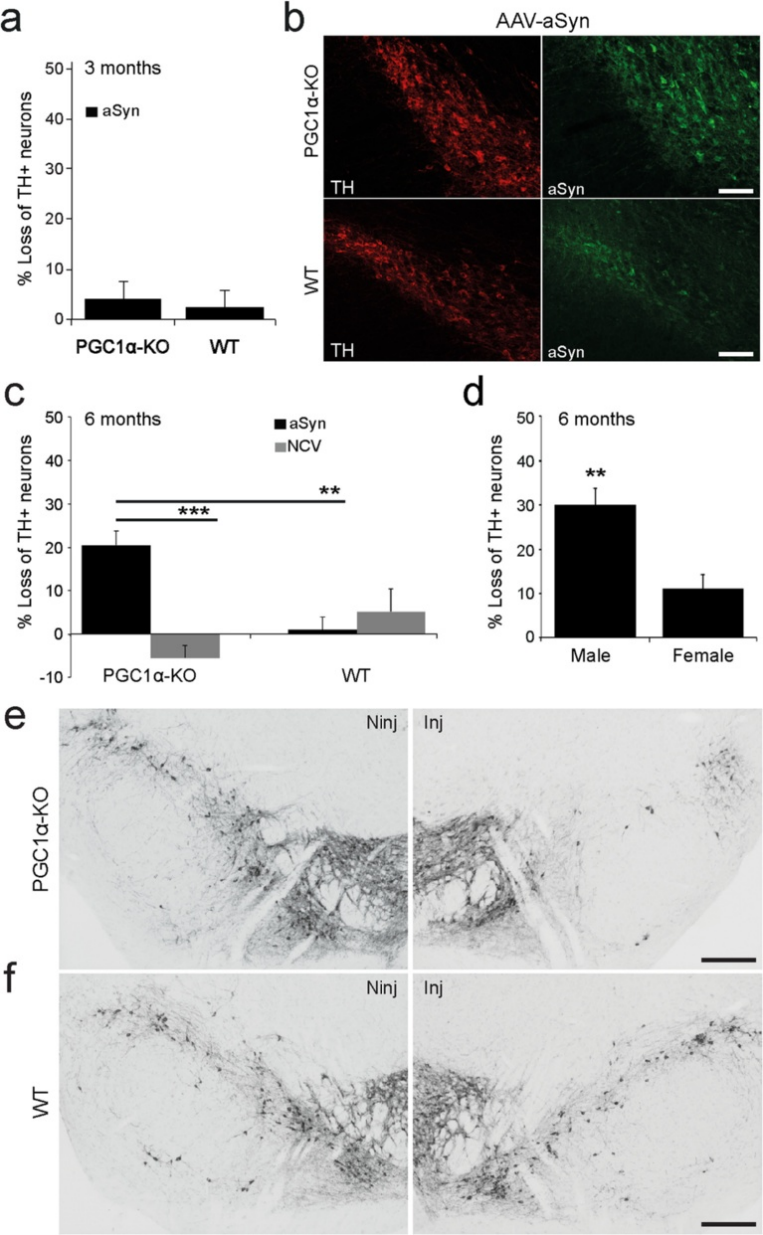
**Expression of human aSyn induces the loss of neurons positive for dopaminergic markers in the SNpc of PGC1α-KO mice.** PGC1α-KO mice or WT mice were injected in the SNpc with an AAV2/6 vector encoding aSyn or a non-coding vector (NCV). **(a)** At 3 months post-injection, there is no significant loss of TH-positive neurons in the SNpc (WT: n = 5 and PGC1α-KO: n = 10). **(b)** Overexpression of human aSyn is detectable by immunohistochemistry in nigral TH-positive neurons. Scale bar: 100 μm. **(c)** At 6 months post-injection, a significant loss of TH-positive neurons is observed in the SNpc of PGC1α-KO mice injected with AAV-aSyn. Statistical analysis: two-way ANOVA with Newman-Keuls post-hoc test; PGC1α-KO + aSyn: n = 18; PGC1α-KO + NCV: n = 5; WT + aSyn: n = 14; WT + NCV: n = 8; **p < 0.01, ***p < 0.001. **(d)** Analysis according to gender reveals that male PGC1α-KO mice are significantly more prone to aSyn-induced loss of TH neurons than female mice. Student’s *t* test: n = 9 for each gender; **p < 0.01. **(e,f)** Representative photomicrographs showing the loss of TH neurons in the AAV-aSyn injected hemisphere of PGC1α-KO mice **(e)**, as compared to no loss in WT mice **(f)**. The non-injected side (NInj) is shown for comparison. Scale bar: 100 μm.

To assess the resistance of nigral dopaminergic neurons to pathologic overabundance of aSyn, an AAV2/6 vector encoding human aSyn (AAV-aSyn) was injected in the SNpc of young adult PGC1α-KO mice. The vector was unilaterally injected, to assess the survival of nigral dopaminergic neurons with respect to the intact, non-injected side. AAV-aSyn was injected at a dose of 1 × 10^7^ TU and compared to the same dose of a control, non-coding vector. Furthermore, age-matched WT C57BL/6J mice were similarly injected and used as control with normal PGC-1α activity. We assessed the number of surviving nigral neurons positive for the dopaminergic marker tyrosine hydroxylase (TH), at 3 and 6 months post-vector injection. At 3 months, there was no loss of dopaminergic neurons, both in WT and PGC1α-KO mice overexpressing human aSyn (Figure 4a). Nevertheless, immunohistochemistry revealed a clear overexpression of human aSyn in nigral TH-positive neurons (Figure 4b).

Remarkably, at 6 months post-injection we measured a 20.5 ± 3.3% loss of TH-positive neurons in the SNpc of PGC1α-KO mice injected with AAV-aSyn, which is significantly different as compared to PGC1α-KO mice injected with the non-coding vector (Figure 4c to e). The loss of neurons was also significantly increased as compared to WT mice injected with AAV-aSyn, in which the SN remained intact (Figure 4c to f). When we compared male and female PGC1α-KO mice injected with AAV-aSyn, we found that the loss of TH-positive neurons was gender-dependent (29.9 ± 3.6% in males versus 11 ± 3.2% in females) (Figure 4d). The loss of dopaminergic neurons was similarly increased in PGC1α-KO mice (29.1 ± 4.1%) compared to WT mice (10.4 ± 6.3%) when measuring the number of neurons positive for the dopamine transporter (DAT) (Student’s *t* test, p = 0.03).

Immunohistochemistry for indicators of apoptotic cell death revealed a positive signal for cleaved caspase-3 only in the SNpc of PGC1α-KO mice injected with the aSyn-expressing vector, on the side ipsilateral to the injection (Additional file 3: Figure S3). To assess the loss of nigrostriatal dopaminergic axons, TH immunoreactivity was further quantified in the striatum. Overall, the loss of striatal TH was below 15% and did not reveal any significant difference between groups (Additional file 4: Figure S4). Indeed, as late as 6 months post-injection, compensatory rescue of striatal dopaminergic innervation could mask the initial partial loss of fibers caused by aSyn overexpression [37].

### Expression of α-synuclein in PGC1α-KO neurons induces the production of reactive oxygen species, which is suppressed by PGC-1α

**Figure 5:**
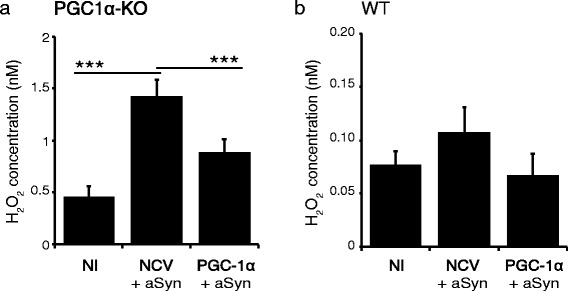
**PGC-1α protects primary neuronal cultures of PGC1α-KO mice against oxidative stress induced by aSyn.** Seven day-old primary neuronal cultures were derived from the cerebral cortex of PGC1α-KO **(a)** or WT mice **(b)**. Individual cultures were co-infected either with the non-coding and aSyn vectors (NCV + aSyn), or with the PGC-1α and aSyn vectors (PGC1α + aSyn). H_2_O_2_ concentrations were measured in cell culture media at 7 days post-infection. Note the significant increase in H_2_O_2_ production in PGC1α-KO neurons expressing aSyn, which is prevented by PGC-1α expression. In contrast, aSyn does not cause any significant increase in H_2_O_2_ production in neurons derived from WT mice. Statistical analysis: one-way ANOVA with Newman-Keuls post-hoc test; NI: n = 14; NCV + aSyn: n = 12; PGC1α + aSyn: n = 14; ***p < 0.001. Note that raw values of H_2_O_2_ production were obtained from separate experiments for neurons derived from PGC1α-KO and WT mice and are therefore not comparable.

PGC1α null mice were previously shown to display a higher sensitivity to oxidative stress, caused by the reduced expression of factors involved in the detoxification of ROS [18]. Hence, to explore possible causes for aSyn toxicity in conditions of low PGC-1α activity, we assessed the production of ROS in the medium of primary neuronal cultures derived from the cortex of either PGC1α-KO mice or WT mice. When 7 day-old neuronal cultures were co-infected with AAV-aSyn in combination with a non-coding control vector, we measured a clear increase in the amount of hydrogen peroxide (H_2_O_2_) produced in the culture medium at 7 days post-infection, as compared to non-infected cultures (Figure 5). Remarkably, co-transduction of neurons with the AAV-PGC-1α vector significantly reduced the production of H_2_O_2_ caused by aSyn expression (Figure 5a). In cortical neurons derived from WT mice, neither the expression of aSyn, nor the co-expression of aSyn and PGC-1α induced any significant change in the production of H_2_O_2_ as compared to non-infected neuronal cultures (Figure 5b). The production of ROS in PGC1α-KO neurons expressing aSyn further highlights the specific neuronal vulnerability to aSyn-induced toxicity in conditions of low PGC-1α activity.

### PGC-1α null neurons expressing human α-synuclein have reduced basal oxygen consumption

**Figure 6:**
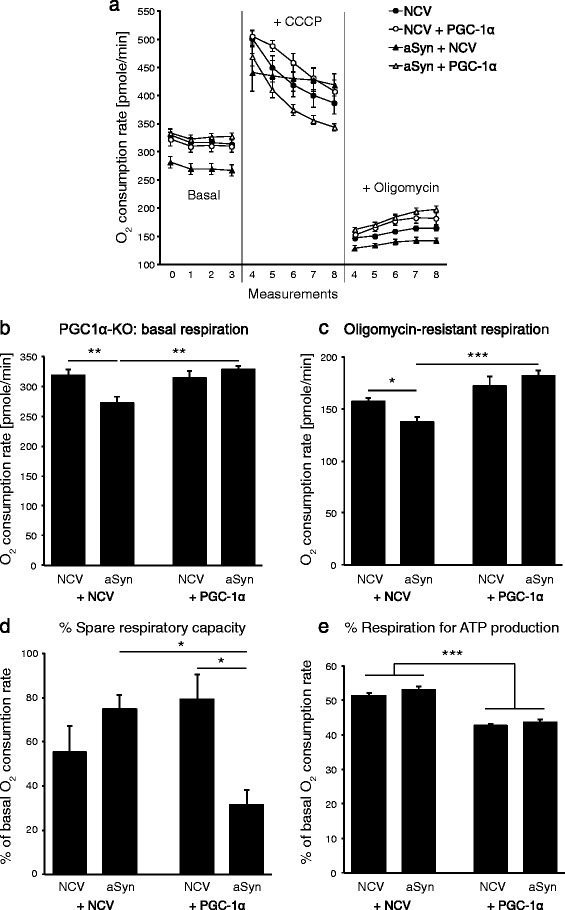
**Alpha-synuclein impairs basal mitochondrial respiration in PGC1α-KO neurons.** Primary neuronal cultures were derived from the cerebral cortex of PGC1α-KO mice and co-transduced with either a non-coding AAV vector (NCV), an AAV vector encoding human aSyn, or with an AAV vector encoding PGC-1α (PGC1α). **(a,b)** Basal oxygen consumption was measured from individual cultures in the conditions NCV alone (n = 15), NCV + aSyn (n = 20), NCV + PGC1α (n = 19) and aSyn + PGC1α (n = 15). **(a,d)** Some of the individual cultures were treated with CCCP, in order to determine the percentage of spare respiratory capacity **(d)**: NCV alone (n = 6), NCV + aSyn (n = 10), NCV + PGC1α (n = 4) and aSyn + PGC1α (n = 5). Other individual cultures were treated with oligomycin to determine **(c)** the oligomycin-resistant residual respiration and **(e)** the percentage of oxygen consumption used for ATP production: NCV alone (n = 9), NCV + aSyn (n = 10), NCV + PGC1α (n = 10) and aSyn + PGC1α (n = 10). Statistical analysis: two-way ANOVA with Newman-Keuls post-hoc test. (b-d): significant interaction between the aSyn and PGC1α effects, *p < 0.05; **p < 0.01; ***p < 0.001; (e): significant group effect of PGC1α, ***p < 0.001.

Next, we used primary cortical neurons derived from PGC1α-KO mice to explore the effect of aSyn on mitochondrial function. Neurons were transduced with AAV expressing either aSyn or PGC-1α. Seven days later, we measured extracellular oxygen flux to evaluate mitochondrial activity. Oxygen consumption rate (OCR) was assessed in basal conditions, in presence of carbonyl cyanide *m*-chlorophenyl hydrazone (CCCP), a compound dissipating the proton gradient across the mitochondrial membrane, and in presence of oligomycin, an inhibitor of the mitochondrial ATP synthase (Figure 6a). We observed that overexpression of aSyn induces a significant decrease in basal OCR in PGC1α-KO neurons (Figure 6b). This effect of aSyn was completely suppressed by the expression of PGC-1α. The changes in OCR appeared very similar in presence of oligomycin, indicating that the reduced respiration found in PGC1α-KO neurons expressing aSyn is not caused by any defect in ATP synthase activity. Therefore, aSyn is likely to lower the overall ETC activity, which can be corrected by restoring PGC-1α expression (Figure 6c).

However, when the maximal respiration rate was measured following addition of the uncoupling agent CCCP (Figure 6a), we found that the spare respiratory capacity was significantly reduced in neurons overexpressing both aSyn and PGC-1α (Figure 6d). Furthermore, the coupling efficiency of the cells, which is indicated by the percentage of basal mitochondrial respiratory rate used for ATP synthase activity, was significantly reduced by PGC-1α expression (Figure 6e). Overall, these results indicate that aSyn perturbs mitochondrial function in PGC1α-KO cells, mainly leading to an impairment of basal respiration which is consistent with the production of ROS observed in these neurons. Expression of PGC-1α rescues this effect, although the respiratory capacity of the neurons remains significantly lower than in neurons that are not expressing aSyn.

### PGC-1α expression protects male PGC1α-KO mice against aSyn toxicity in the SNpc

**Figure 7:**
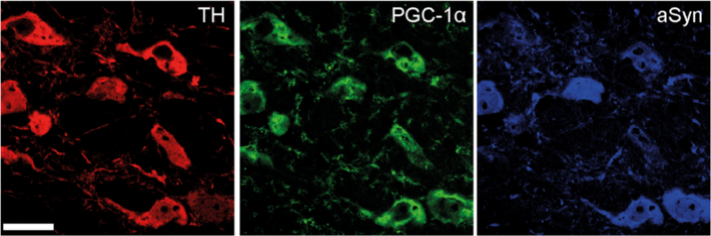
**Co-injection of AAV-aSyn and AAV-PGC-1α vectors induces expression of PGC-1α and aSyn in nigral neurons.** Immunostaining shows the co-expression of PGC-1α and aSyn in TH-positive neurons in the SNpc of PGC1α-KO mice, at 6 months post-AAV injection. Scale bar: 20 μm.

The significant protective effect observed with the AAV-PGC-1α vector prompted further investigation to determine if the same vector could prevent neuronal degeneration *in vivo*. To address this question, we assessed if injection of the AAV-PGC-1α vector in the SNpc of PGC1α-KO mice could protect nigral neurons against aSyn toxicity. As intranigral injection of the AAV-aSyn vector produced only mild neurodegenerative effects in our previous experiment, we used another vector design including an optimized kozak sequence and the WPRE enhancer element to increase the rate of aSyn translation. In a previous study, this vector was found to express higher levels of aSyn in the rat SNpc [26]. We first assessed if the co-expression of aSyn and PGC-1α could be achieved on the long-term following co-injection of the two vector suspensions. Indeed, we could detect by immunohistochemistry a clear overexpression of both mouse PGC-1α and human aSyn in a significant proportion of TH-labeled dopaminergic neurons, 6 months post-injection of the vector mix (Figure 7).

**Figure 8:**
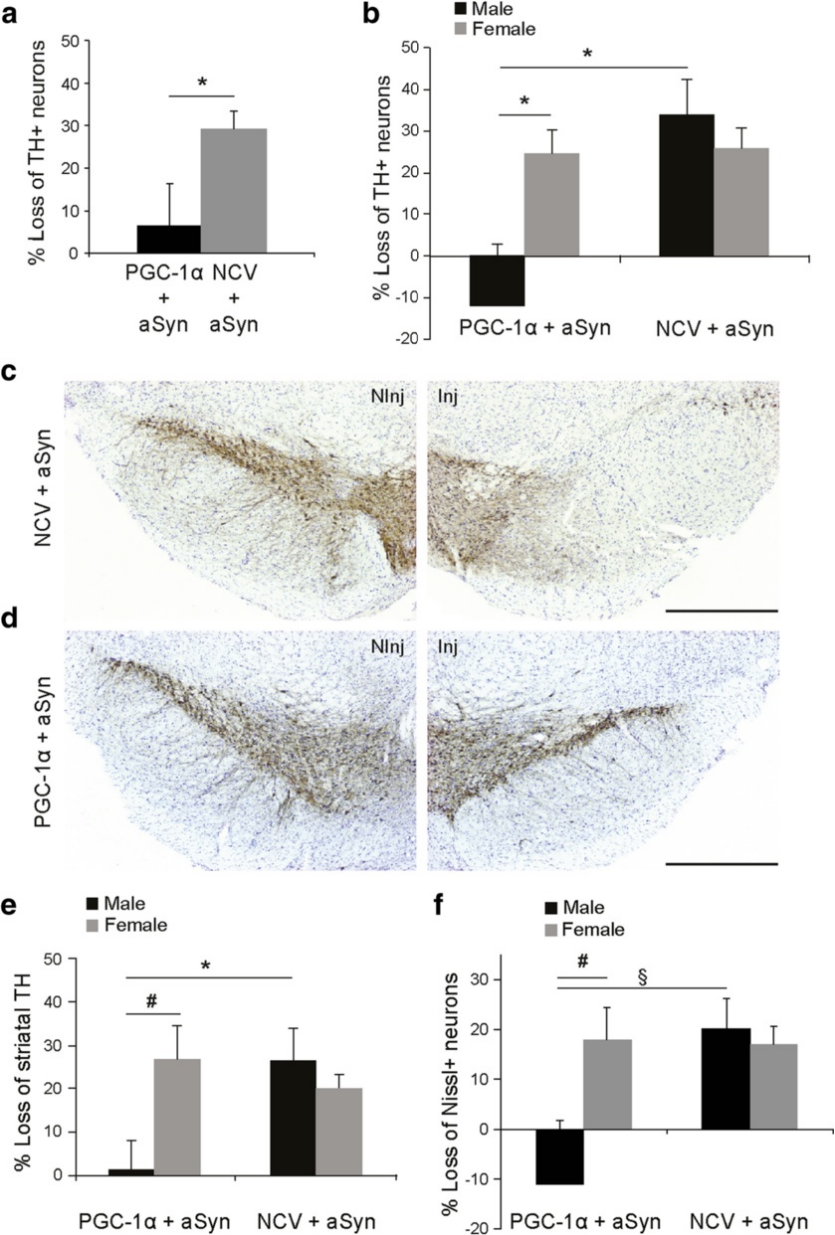
**PGC-1α expression protects against aSyn toxicity in the SNpc of male PGC1α-KO mice.** PGC1α-KO mice were co-injected with two AAV2/6 vectors encoding for aSyn and PGC-1α (PGC1α + aSyn). The control group is injected with a non-coding vector instead of AAV-PGC-1α (NCV + aSyn). **(a)** Loss of TH-positive neurons in the SNpc at 6 months post-injection. PGC-1α overexpression induces significant protection against aSyn toxicity. Statistical analysis: Student’s *t* test; PGC1α + aSyn: n = 10; NCV + aSyn: n = 10; *p < 0.05. **(b)** Analysis according to gender shows that the protective effect of AAV-PGC-1α is specific to male mice. Statistical analysis: two-way ANOVA with Newman-Keuls post-hoc test; PGC1α + aSyn: n = 4 males and 6 females; NCV + aSyn: n = 5 males and 5 females; *p < 0.05. **(c,d)** Representative photomicrographs showing the loss of TH-positive neurons in the SNpc of NCV + aSyn mice **(c)**, as compared to PGC1α + aSyn mice **(d)**. The non-injected side (NInj) is shown for comparison. Scale bar: 500 μm. **(e)** Gender-specific analysis reveals significant protection of striatal TH fibers only in male mice PGC1α-KO mice injected with the AAV-PGC-1α vector. Statistical analysis as in (b); *p < 0.05 and #p = 0.058. **(f)** Stereological gender-specific analysis of the loss of Nissl-positive neurons in the SNpc. Statistical analysis as in (b): # p = 0.070, § p = 0.059.

A gender-balanced cohort of young adult PGC1α-KO mice was co-injected in the SNpc with the optimized AAV2/6 vector encoding aSyn (1 × 10^7^ TU), together with a control non-coding vector (5 × 10^6^ TU). Six months post-injection, PGC1α-KO mice indeed showed a significant, 29.1 ± 4.3% loss of nigral TH-positive neurons in the ipsilateral SNpc (Figure 8a to c). In male mice, nigral degeneration induced by the optimized vector reached 34 ± 8.4% loss of TH-positive neurons, as compared to the non-injected side (Figure 8b). In female mice, the extent of dopaminergic neuron loss was slightly lower (25.8 ± 4.8%). With the optimized aSyn-expressing vector, neuronal loss in female mice was approximately twice higher than in our previous experiment (see Figure 4d), no more significantly different from their male counterparts. In the striatum, the effect on dopaminergic axons was also more pronounced. We measured an average loss of TH immunoreactivity of 22.7 ± 3.4% over the whole striatum, reaching 26.4 ± 7.4% in male, and 20.2 ± 3.1% in female mice (Figure 8e). To verify if the loss of the dopaminergic marker TH was indeed associated with neuronal degeneration, we performed stereological estimates of the number of Nissl-stained neuronal nuclei in the SNpc. We found an overall 18.7 ± 3.0% reduction in the number of neuronal nuclei in the injected SNpc (18.0 ± 6.0% in male and 17.1 ± 3.6% in female mice), consistent with aSyn-induced neuronal loss (Figure 8f).

Next, we assessed the effect of PGC-1α expression on nigral neurons. The co-injection of the AAV-PGC-1α vector (5 × 10^6^ TU) showed protective effects against degeneration induced by the expression of human aSyn in nigral dopaminergic neurons (Figure 8a to d). As compared to the 29.1 ± 4.3% loss of TH-positive neurons in PGC1α-KO mice injected with AAV-aSyn, we found a significantly reduced neuronal loss (6.4 ± 9.8%, p < 0.05) following co-injection with AAV-PGC-1α (Figure 8a). When we compared the effect of PGC-1α between genders, PGC-1α expression completely rescued TH-positive nigral neurons from aSyn toxicity in males, but did not show any effect in female mice (Figure 8b). Analysis of striatal TH immunoreactivity confirmed the neuroprotection observed in male mice only (Figure 8e). Finally, there was a trend towards higher number of Nissl-positive nuclei in the SNpc of male mice injected with AAV-PGC-1α, suggesting that expression of PGC-1α was able to rescue neurons from aSyn toxicity (Figure 8f).

**Figure 9:**
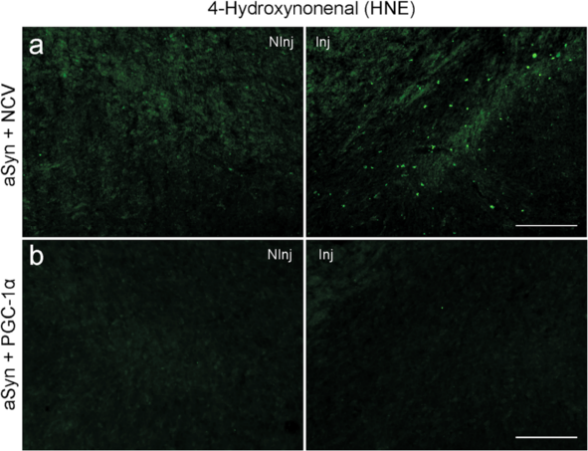
**PGC-1α prevents oxidative stress induced by aSyn** ***in vivo.*** **(a)** Immunostaining for HNE, a marker for oxidative damage, at 6 months after co-injection of PGC1α-KO mice with an AAV2/6 vector encoding aSyn and a control non-coding vector (NCV). Note the presence of the HNE staining in the SNpc of PGC1α-KO mice expressing aSyn. **(b)** Co-injection with AAV2/6 vectors encoding aSyn and PGC-1α. Note that PGC-1α overexpression suppresses signs of oxidative stress in PGC1α-KO mice expressing aSyn. The non-injected side (NInj) is shown for comparison. Scale bar: 100 μm.

As we previously found a major effect of PGC-1α on the production of ROS, we analyzed the accumulation of 4-hydroxynonenal (HNE), a histological marker for lipid peroxidation, in the SNpc of these animals. HNE deposition was detected in the SNpc of male PGC1α-KO mice, on the side expressing human aSyn (Figure 9a). In contrast, when AAV-PGC-1α was co-injected with the aSyn-expressing vector, the HNE signal was completely suppressed, further demonstrating the protective effect of restoring PGC-1α activity on cellular damage caused by ROS production (Figure 9b).

Altogether, these results underline the neuroprotective effect of PGC-1α against the toxic effect of aSyn accumulation. Particularly in male mice, the loss of PGC-1α activity prompts neuronal loss when aSyn is chronically expressed in the SNpc, highlighting the role of this transcriptional co-activator in gender-dependent neuronal resistance to degeneration.

## Discussion

Age-related neurodegenerative diseases are often characterized by downregulated PGC-1α activity and perturbed expression of PGC-1α transcriptional targets [38-40,21,41]. This transcriptional co-regulator has therefore emerged as a potential therapeutic target to oppose neuronal degeneration in PD. Here, we show that nigral dopaminergic neurons of PGC-1α null mice, which presumably mirror some aspects of premature brain aging, display profound ultrastructural alterations of ER and mitochondria. Furthermore, reduced PGC-1α activity leads to increased vulnerability to aSyn, specifically in male mice. This effect is associated with increased oxidative damage, consistent with the role of PGC-1α in mitochondrial function and ROS detoxification. In this context, expression of full-length PGC-1α using an AAV vector can rescue aSyn-induced neurodegeneration in male mice.

To determine how reduced PGC-1α activity affects nigral neurons, we analyzed changes in mitochondrial morphology and density. Despite the transcriptional role of PGC-1α in mitochondrial biogenesis, we do not find any major decrease in the density of mitochondria, neither in the striatal dopaminergic axons, nor in the neuronal soma located in the SN. Therefore, dopaminergic neurons are likely to compensate for the loss of full-length PGC-1α, possibly via other PGC-1 variants. A mutant form of NT-PGC-1α is expressed in PGC1α-KO mice and could potentially control mitochondrial biogenesis in the absence of full-length PGC-1α [29]. However, this factor has been shown to regulate a gene program that is clearly distinct from full-length PGC-1α [42]. Instead, PGC-1β could support mitochondrial biogenesis [31]. Although PGC-1β is non-significantly increased in the SN of PGC1α-KO mice, its expression is reduced when PGC-1α is overexpressed, demonstrating a relationship in the activity of these two transcriptional co-factors (Figure 1b). PGC-1β is involved in basal mitochondrial biogenesis, whereas the role of PGC-1α is rather to adapt cell metabolic activity, for instance by inducing mitochondrial proliferation in mitochondrial disease [43,44]. Remarkably, myocytes lacking both PGC-1α and β show no difference in mitochondrial content, despite a clear reduction in ETC activity, suggesting that these two parameters are not necessarily coupled, and that PGC-1 activity is primarily involved in adapting oxidative capacity [45].

PGC1α-KO mice display clear alterations in mitochondrial morphology. In particular, giant mitochondria are observed in nigral neurons of 10 month-old PGC1α-KO mice, while they are nearly absent from WT mice. In the absence of PGC-1α, PGC-1β might promote mitochondrial fusion and elongate mitochondrial tubules [46]. Alternatively, the accumulation of giant mitochondria could be due to a selective reduction in mitochondrial fission. Abnormally large mitochondria are indicative of organelle senescence, often accompanied by structural alterations such as swelling, partial loss *cristae*, and almost complete destruction of ETC components [47]. These structural changes underlie functional deficiencies such as disturbed energy metabolism, and subsequent decrease in ATP production, often associated with age-dependent mitochondrial DNA mutations [48]. The accumulation of giant mitochondria in nigral dopaminergic neurons of PGC1α-KO mice may therefore be a sign of premature neuronal aging.

Conversely, injection of the AAV-PGC-1α vector in the SNpc produces clear changes in mitochondrial dynamics. The size and clustering of mitochondria are both reduced, consistent with increased mitochondrial fission. PGC-1α controls the expression of the GTPases dynamin-related protein 1 (Drp1) and mitofusin 2 (Mfn2) in human skeletal muscle and mouse ventral midbrain neurons [19,49]. These factors have critical roles in mitochondrial fission (Drp1) and fusion (Mfn2), regulating tubular mitochondrial networks. Defects in fission or fusion may cause neuronal death by limiting mitochondrial motility, decreasing energy production, impairing Ca^2+^ buffering, and promoting oxidative stress and mtDNA deletion [50]. Overall, the expression of PGC-1α in adult nigral neurons can suppress the signs of premature mitochondrial senescence that are found in PGC1α-KO mice.

Tightly related to the aging process, mitochondrial turnover is essential to replace damaged mitochondria with functional, replicating organelles. Several indicators suggest that the rate of mitochondrial turnover is affected by PGC-1α in nigral dopaminergic neurons. A significant increase in the number of mitochondria, together with a low density of lipofuscin granules, a by-product of autophagic degradation, indicate a low rate of mitochondrial turnover in PGC1α-KO mice. Indeed, autophagic elimination of giant mitochondria could be further compromised by the large size of these organelles [51]. Conversely, neuronal soma over-expressing PGC-1α display small-sized mitochondria, together with enhanced accumulation of lipofuscin granules, consistent with high mitochondrial turnover.

The ER is an organelle closely associated to mitochondria, which may have important function in mitochondrial dynamics including fission events [52]. The interface between ER and mitochondria is called the mitochondria-associated ER membrane (MAM), which has critical roles in Ca^2+^ signaling and lipid exchange between organelles. In PGC1α-KO mice, we observe disrupted ER morphology, with abundant free ribosomes and scattered, fragmented cisternae, which have lost their parallel stack arrangement. PGC-1α increases the number of membrane contacts between the ER and the mitochondria (Figure 3f). Notably, aSyn has been recently localized in the MAM, with pathogenic mutations affecting mitochondrial dynamics [53].

Here, we find that the loss of PGC-1α enhances the vulnerability of dopaminergic neurons to aSyn toxicity in the SNpc. Alpha-synuclein has been shown to interfere with a number of cellular processes including membrane trafficking [54], autophagy [55], proteasomal degradation [56] and mitochondrial function [57]. Alpha-synuclein can directly affect mitochondrial morphology, by interacting with mitochondrial membranes containing cardiolipin and promoting mitochondrial fragmentation [12,11]. Moreover, aSyn can contribute to neurodegeneration by inducing ER stress [58,59], which may further precipitate mitochondrial perturbations. A few reports have characterized the mitochondrial dysfunction caused by the accumulation of aSyn. The protein has been reported to interact with complex I, interfere with its function and lead to increased ROS production [60,61]. Alpha- and beta-synucleins were recently reported to reduce basal respiration in primary neurons [62]. We find that aSyn expression in PGC-1α null neurons leads to a significant decrease in mitochondrial respiration, consistent with the reported effects of aSyn on ETC. activity. Remarkably, the aSyn-induced decrease in mitochondrial respiration can be rescued by *de novo* expression of PGC-1α. However, PGC-1α expression also decreased the reserve respiratory capacity of the neuronal cells, similar to previous results obtained in neurons derived from the ventral midbrain [19]. It will be important to explore the consequences of PGC-1α on long-term neuronal viability, as the capacity of the cells to adapt their mitochondrial respiration could be important to meet with high ATP demand. Nevertheless, these results suggest that reduced PGC-1α activity can contribute to the mitochondrial dysfunction caused by aSyn. It is also possible that other mechanisms, independent from the mitochondrial function, are the cause of the increased vulnerability of PGC-1α-null neurons accumulating aSyn, as suggested by a recent study which found signs of enhanced aSyn oligomerization [22].

Chronic overexpression of PGC-1α has previously been reported to decrease the expression of dopaminergic markers in the adult SN, consistent with downregulation of the transcription factor Pitx3 [19,23]. We found no such evidence in PGC1α-KO mice. This is surprising as the injection of the AAV-PGC-1α vector leads to a 10-fold higher level of PGC-1α mRNA compared to WT mice (Figure 1a). It is plausible that local PGC-1α overexpression does not produce the same effects in the normal SN and in the context of a PGC-1α-null brain, where other cells still fail to express this protein. Remarkably, and in contrast to previous experiments using WT neurons, we did not find any significant increase in basal respiration in PGC1α-KO neurons overexpressing PGC-1α (Figure 6b), suggesting that these neurons may also have developed compensatory mechanisms to regulate mitochondrial function in the absence of PGC-1α. Altogether, these results suggest that in conditions where PGC-1α is downregulated, nigral dopaminergic neurons may better tolerate therapeutic strategies that enhance PGC-1α activity, even in a chronic manner.

Enhanced vulnerability to aSyn, as well as the rescue effect of overexpressing PGC-1α, was predominantly observed in male PGC1α-KO mice. PGC-1α has also been identified as a male-specific disease modifier of human and experimental amyotrophic lateral sclerosis [63]. This gender effect is reminiscent of the higher incidence of PD in men than in women [64]. Studies on gene expression in nigral dopaminergic neurons from healthy control and PD subjects have shown differences between males and females [65], which suggests that transcriptional activity of specific set of genes could be an important factor in the male vulnerability to disease. Remarkably, a set of genes associated to mitochondrial function appears to be deregulated mainly in male PD cases. It will be critical to determine if PGC-1α activity is involved in this difference. Another possible explanation for the sexual dimorphism observed in PGC1α-KO mice may lie in the response to sex hormones. Indeed, expression of the estrogen receptor α (ERα) is linked to PGC-1α activity [66]. Although the neuroprotective effects of estrogens is still debated in PD [67], it is possible that expression of estrogen receptors is reduced in PGC1α-KO mice, increasing the vulnerability of male mice to pathogenic factors. Mitochondrial activity may also have a critical role in gender asymmetry. As the mitochondrial genome is transmitted through the maternal lineage, deleterious mitochondrial DNA (mtDNA) mutations that affect only males will not be subject to natural selection, possibly contributing to the sexual dimorphism observed in aging or neurodegeneration [68].

There is increasing evidence for the critical role of PGC-1α in PD, prompting further investigation on the therapeutic potential of modulating its activity. Pioglitazone, an agonist of peroxisome proliferator-activated receptor-γ (PPARγ) promoting PGC-1α activity, is notably neuroprotective in the MPTP model of PD [69-71]. A phase II clinical trial is currently conducted to assess the effect of pioglitazone in early PD.

## Conclusions

Using a genetic aSynbased approach, the present study further highlights the protective role of PGC-1α in nigral dopaminergic neurons. Preserving PGC-1α activity in the SNpc may prevent age-related alterations underlying neuronal vulnerability in PD. In addition, our results indicate a role for PGC-1α in the effect of sex and age on the risk of developing PD. Further elucidating the neuroprotective role of PGC-1α might lead to the development of novel therapeutic strategies.

## Availability of supporting data

The data sets supporting the results of this article are included within the article (and its additional files).

## Additional files

Additional file 1: figure S1. PGC1α-KO mice have normal density of mitochondria in the striatal dopaminergic axons. **(a)** MitoDsRed fluorescence colocalizing with TH-positive neurons in the substantia nigra of a 10 months-old mouse injected with the AAV-mitoDsRed vector. Scale bar: 200 μm. **(b)** MitoDsRed fluorescence colocalizing with TH-positive axonal fibers in the dorsolateral striatum of a mouse injected with the AAV-mitoDsRed vector. Scale bar: 100 μm. **(c)** Relative quantification of the number of mitoDsRed-positive discrete particles normalized to the area of TH immunoreactivity in randomly selected regions of the dorsolateral striatum. Data points are indicated on the bar graph for each mouse from the WT (n = 4) and PGC1α-KO (n = 5) cohorts. Open squares represent male mice and open circles represent female mice in each group. Statistical analysis: two-tailed Student’s *t* test with equal variance; ns: non-significant (p = 0.32). Additional file 2: figure S2. Loss of PGC1-1α does not cause any major abnormal mitochondrial phenotype in neuronal cells localized in the VTA. **(a)** In neuronal cells of the VTA, PGC1α-KO and WT mice display similar mitochondrial density. **(b)** Average surface of mitochondrial area in PGC1α-KO and WT mice. **(c)** Nearest neighbor analysis of the minimal distance between mitochondria showing a similar distribution of the organelles in both groups. **(d)** Accumulation of lipofuscin granules in the VTA of PGC1α-KO and WT mice. Statistical analysis: Student’s *t* test with equal variance; (b) WT n = 4110 mitochondria; PGC1α-KO n = 7265 mitochondria; (A,C,D): WT n = 51 neurons; PGC1α-KO n = 95 neurons; ***p < 0.001. Micrographs were obtained from 3 animals in each group. Additional file 3: figure S3. Overexpression of aSyn leads to detectable cleaved Caspase 3 signal in PGC1α-KO mice. **(a**) Positive signal for cleaved Caspase 3 signal is detected in the hemisphere injected with AAV-aSyn in the SNpc of PGC1α-KO mice. **(b)** No positive immunostaining for cleaved Caspase 3 can be detected in WT mice at 6 months post-injection. The non-injected side is shown for comparison. Scale bar: 100 μm. Additional file 4: figure S4. Loss of dopaminergic axons in the striatum of PGC1α-KO and WT mice overexpressing aSyn. PGC1α-KO mice or WT mice were injected in the SNpc with an AAV2/6 vector encoding aSyn or a non-coding vector (NCV). **(a)** No significant difference between groups is found in the striatal density of TH-positive fibers. Statistical analysis: two-way ANOVA with Newman-Keuls post-hoc test; PGC1α-KO + aSyn: n = 18; PGC1α-KO + NCV: n = 5; WT + aSyn: n = 14; WT + NCV: n = 8. **(b)** Although the loss of striatal TH density tends to be higher in male PGC1α-KO mice overexpressing aSyn, there is no significant difference between genders. Statistical analysis: Student’s *t* test with equal variance; n = 9 for male and female mice.
