# Supplementary figures and images for: PGC-1α activity in nigral dopamine neurons determines vulnerability to α-synuclein
Source: Acta Neuropathol Commun. 2015 Apr 1;3:16. doi: 10.1186/s40478-015-0200-8 (PMC4379693; doi:10.1186/s40478-015-0200-8)

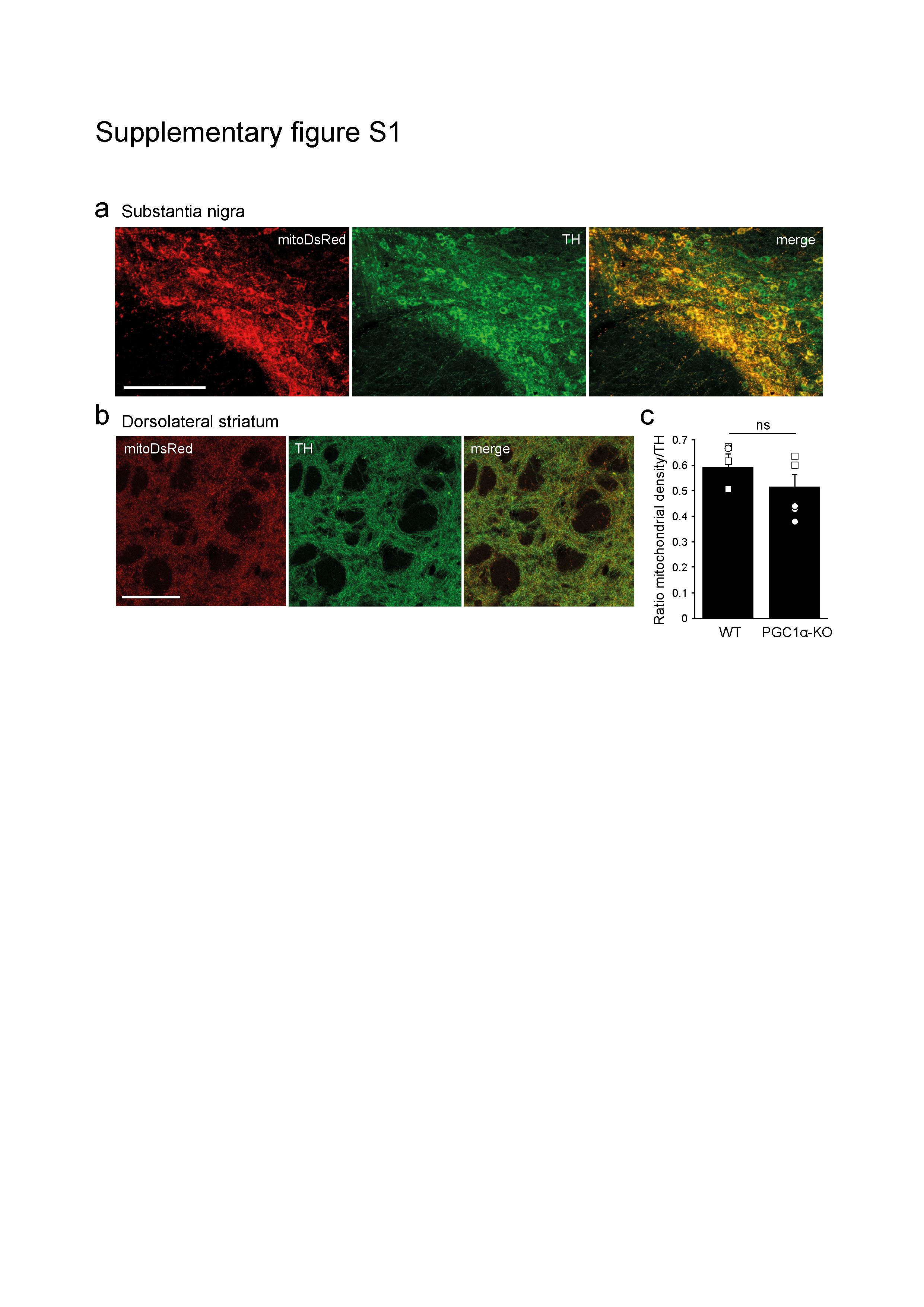

Supplement: Additional file 1: figure S1. — PGC1α-KO mice have normal density of mitochondria in the striatal dopaminergic axons. (a) MitoDsRed fluorescence colocalizing with TH-positive neurons in the substantia nigra of a 10 months-old mouse injected with the AAV-mitoDsRed vector. Scale bar: 200 μm. (b) MitoDsRed fluorescence colocalizing with TH-positive axonal fibers in the dorsolateral striatum of a mouse injected with the AAV-mitoDsRed vector. Scale bar: 100 μm. (c) Relative quantification of the number of mitoDsRed-positive discrete particles normalized to the area of TH immunoreactivity in randomly selected regions of the dorsolateral striatum. Data points are indicated on the bar graph for each mouse from the WT (n = 4) and PGC1α-KO (n = 5) cohorts. Open squares represent male mice and open circles represent female mice in each group. Statistical analysis: two-tailed Student’s t test with equal variance; ns: non-significant (p = 0.32). [file 40478_2015_200_MOESM1_ESM.tif]

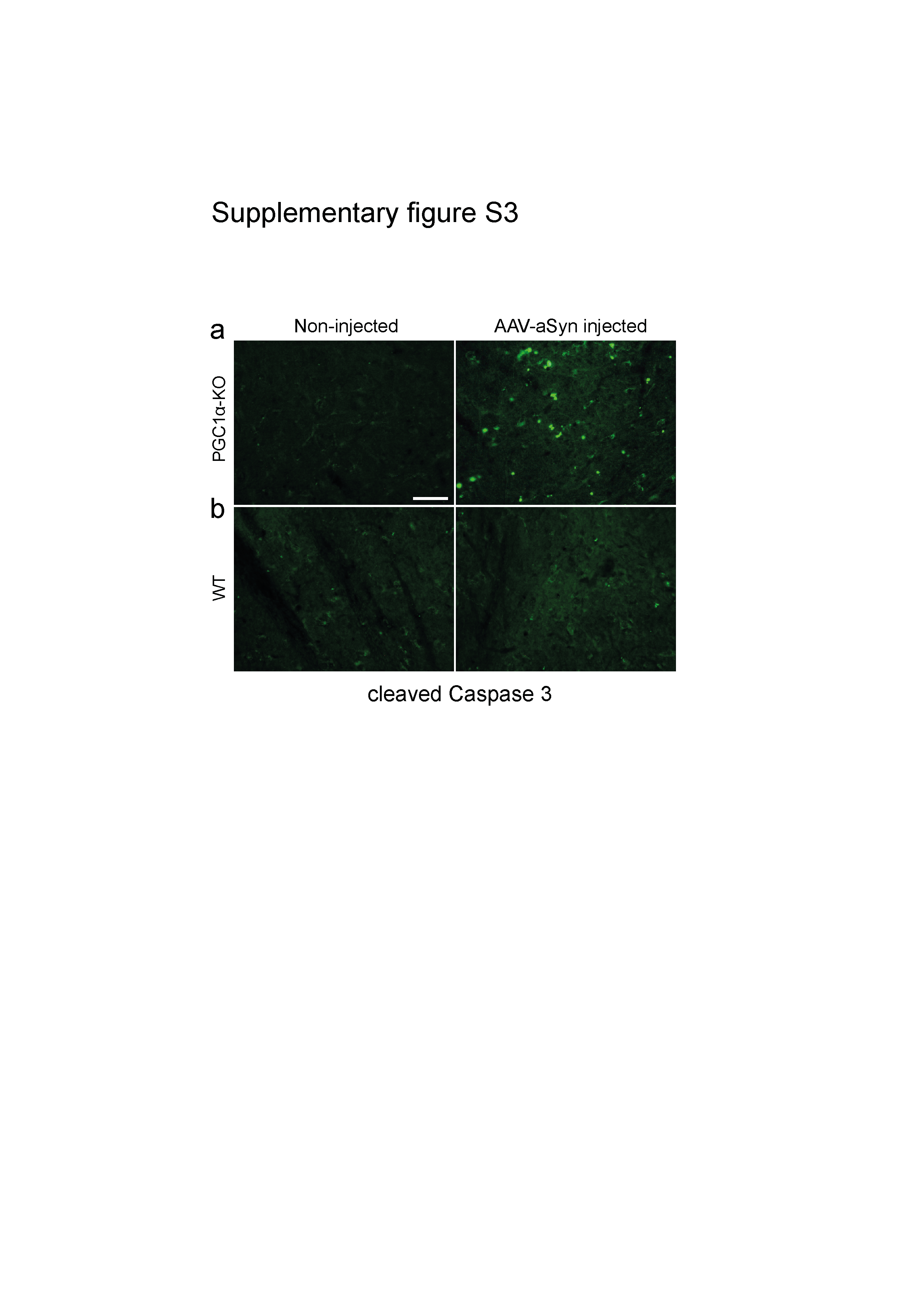

Supplement: Additional file 3: figure S3. — Overexpression of aSyn leads to detectable cleaved Caspase 3 signal in PGC1α-KO mice. (a) Positive signal for cleaved Caspase 3 signal is detected in the hemisphere injected with AAV-aSyn in the SNpc of PGC1α-KO mice. (b) No positive immunostaining for cleaved Caspase 3 can be detected in WT mice at 6 months post-injection. The non-injected side is shown for comparison. Scale bar: 100 μm. [file 40478_2015_200_MOESM3_ESM.tif]

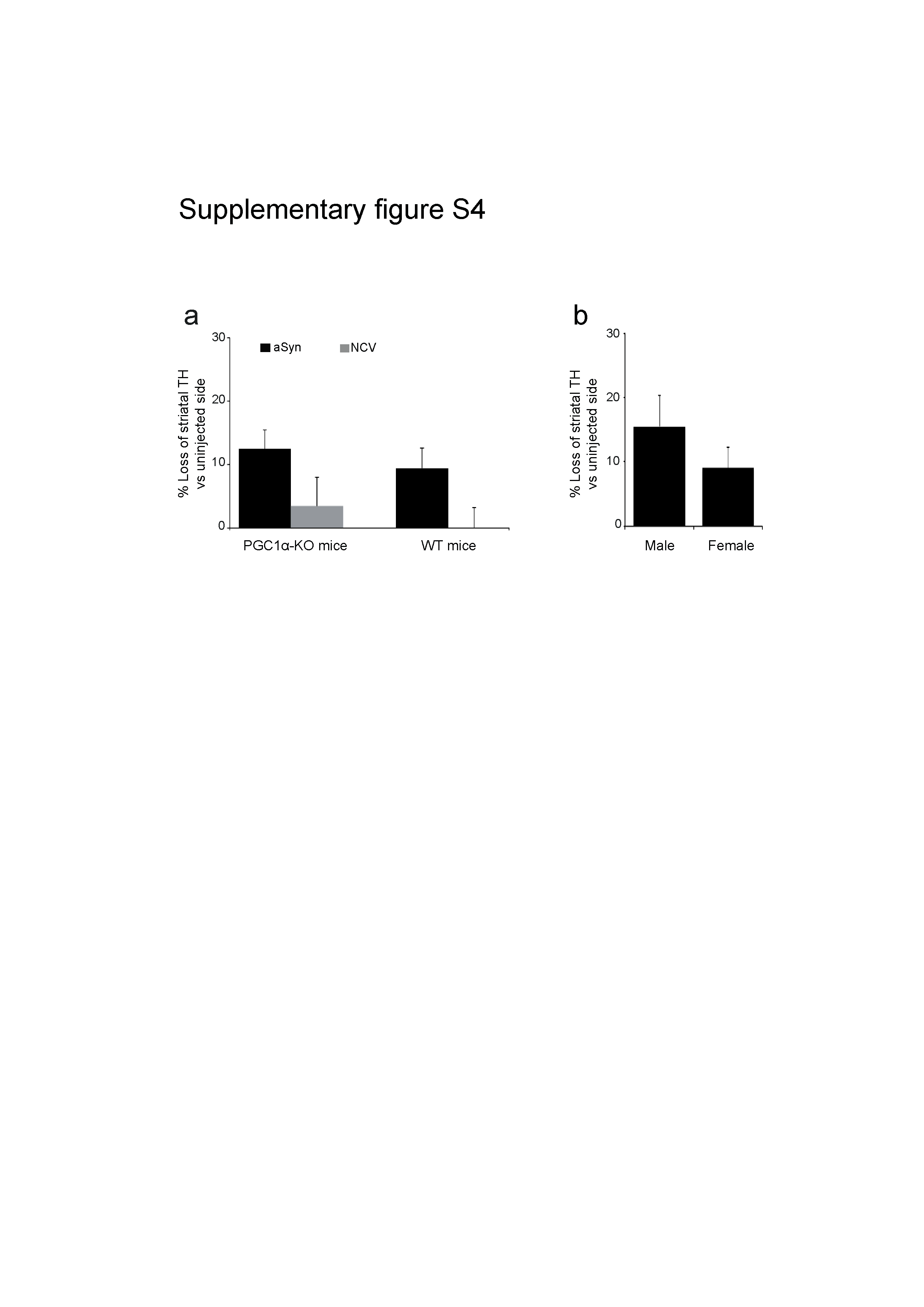

Supplement: Additional file 4: figure S4. — Loss of dopaminergic axons in the striatum of PGC1α-KO and WT mice overexpressing aSyn. PGC1α-KO mice or WT mice were injected in the SNpc with an AAV2/6 vector encoding aSyn or a non-coding vector (NCV). (a) No significant difference between groups is found in the striatal density of TH-positive fibers. Statistical analysis: two-way ANOVA with Newman-Keuls post-hoc test; PGC1α-KO + aSyn: n = 18; PGC1α-KO + NCV: n = 5; WT + aSyn: n = 14; WT + NCV: n = 8. (b) Although the loss of striatal TH density tends to be higher in male PGC1α-KO mice overexpressing aSyn, there is no significant difference between genders. Statistical analysis: Student’s t test with equal variance; n = 9 for male and female mice. [file 40478_2015_200_MOESM4_ESM.tif]
